# An Integrative Machine Learning Model for Predicting Early Safety Outcomes in Patients Undergoing Transcatheter Aortic Valve Implantation

**DOI:** 10.3390/medicina61030374

**Published:** 2025-02-21

**Authors:** Abilkhair Kurmanaliyev, Kristina Sutiene, Rima Braukylienė, Ali Aldujeli, Martynas Jurenas, Rugile Kregzdyte, Laurynas Braukyla, Rassul Zhumagaliyev, Serik Aitaliyev, Nurlan Zhanabayev, Rauan Botabayeva, Yerlan Orazymbetov, Ramunas Unikas

**Affiliations:** 1 Department of Cardiology, Hospital of Lithuanian University of Health Sciences Kauno Klinikos, Lithuanian University of Health Sciences, 2 Eivenių Str., LT-50009 Kaunas, Lithuania; abilxaer1@gmail.com (A.K.); rima.braukyliene@lsmu.lt (R.B.); ali.aldujeli@universityofgalway.ie (A.A.); martynas.jurenas@kaunoklinikos.lt (M.J.); rugile.kregzdyte@stud.lsmu.lt (R.K.); laurynas.braukyla@stud.lsmu.lt (L.B.); ramunas.unikas@yahoo.com (R.U.); 2 Department of Mathematical Modeling, Kaunas University of Technology, Studentų Str. 50–143, LT-50009 Kaunas, Lithuania; kristina.sutiene@ktu.lt; 3 CORRIB Research Centre for Advanced Imaging and Core Laboratory, University of Galway, 1 University Road Str., H91 TK33 Galway, Ireland; 4 Department of Cardiac, Thoracic and Vascular Surgery, Hospital of Lithuanian University of Health Sciences Kauno Klinikos, Lithuanian University of Health Sciences, 2 Eivenių Str., LT-50009 Kaunas, Lithuania; rasszhum0425@kmu.lt (R.Z.); doctoryerlan@gmail.com (Y.O.); 5 Faculty of Medicine and Health Care, Al-Farabi Kazakh National University, 71 Al-Farabi Ave., Almaty 050040, Kazakhstan; 6 South Kazakhstan Medical Academy, 1 Al-Farabi Square, Shymkent 160019, Kazakhstan; nur-7979@mail.ru (N.Z.); rauana.ex@mail.ru (R.B.); 7 National Scientific Medical Center, 42 Abylaikhan Avenue, Astana 010009, Kazakhstan

**Keywords:** aortic stenosis, transcatheter aortic valve implantation, early safety outcomes, ADASYN, random forest, SHAP

## Abstract

*Background and Objectives*: Early safety outcomes following transcatheter aortic valve implantation (TAVI) for severe aortic stenosis are critical for patient prognosis. Accurate prediction of adverse events can enhance patient management and improve outcomes. *Materials and Methods*: This study aimed to develop a machine learning model to predict early safety outcomes in patients with severe aortic stenosis undergoing TAVI. We conducted a retrospective single-centre study involving 224 patients with severe aortic stenosis who underwent TAVI. Seventy-seven clinical and biochemical variables were collected for analysis. To handle unbalanced classification problems, an adaptive synthetic (ADASYN) sampling approach was used. A fined-tuned random forest (RF) machine learning model was developed to predict early safety outcomes, defined as all-cause mortality, stroke, life-threatening bleeding, acute kidney injury (stage 2 or 3), coronary artery obstruction requiring intervention, major vascular complications, and valve-related dysfunction requiring repeat procedures. Shapley Additive Explanations (SHAPs) were used to explain the output of the machine learning model by attributing each variable’s contribution to the final prediction of early safety outcomes. *Results*: The random forest model identified left femoral artery diameter and aortic valve calcification volume as the most influential predictors of early safety outcomes. SHAPs analysis demonstrated that smaller left femoral artery diameter and higher aortic valve calcification volume were associated with poorer early safety prognoses. *Conclusions*: The machine learning model highlights of early safety outcomes after TAVI. These findings suggest that incorporating these variables into pre-procedural assessments may improve risk stratification and inform clinical decision-making to enhance patient care.

## 1. Introduction

Aortic stenosis (AS), a prevalent valvular heart condition in older adults over 65 years of age, affects approximately 2–7% of this population and is further exacerbated by risk factors like hypertension, diabetes, and hyperlipidemia [1,2]. Untreated AS poses significant morbidity and mortality risks [3]. However, due to elevated surgical risks, many patients are not eligible for surgical aortic valve replacement (SAVR) [4,5]. For such high-risk groups, transcatheter aortic valve implantation (TAVI) has emerged as a minimally invasive alternative, offering effective treatment for severe symptomatic AS while avoiding open surgery [6,7].

Despite its advantages, predicting TAVI outcomes is complex. Postoperative results are influenced by multiple factors, including patient comorbidities, anatomical features, and preoperative health conditions [8,9]. This variability underscores the need for precise risk stratification and individualized treatment approaches [10].

Machine learning (ML) methods have transformed cardiovascular medicine by improving predictive models for clinical outcomes through the detection of intricate, non-linear variable relationships [11,12]. In the context of TAVI, ML approaches have surpassed traditional statistical methods in predicting complications such as vascular injury, valve dysfunction, and acute kidney injury [13,14]. Random forest algorithms, for instance, effectively classify patient risks by integrating diverse clinical and imaging data [15,16].

Advanced imaging modalities, such as computed tomography (CT), are essential for preoperative planning in TAVI, providing critical parameters like aortic valve calcification volume, left ventricular outflow tract dimensions, and femoral artery diameter, which are strong predictors of procedural success and complications [17,18]. Combining echocardiographic findings with ML algorithms further enhances predictive accuracy [19,20].

This study leverages an integrative ML framework to identify predictors of early safety outcomes in TAVI patients. Employing adaptive synthetic (ADASYN) sampling to address data imbalance and SHapley Additive exPlanations (SHAPs) for interpretability, this research provides a robust approach for personalized risk stratification [21,22]. These methods, validated across clinical applications, improve prediction reliability and decision-making utility [23,24].

Advancements in TAVI device technology, including balloon-expandable and self-expanding prostheses, have significantly enhanced procedural success rates [25,26]. However, these innovations necessitate detailed preoperative evaluations to mitigate risks associated with challenging anatomical conditions, such as severe calcification and narrow vascular access [27,28].

This research aims to bridge the gap between advanced ML techniques and clinical practice by utilizing a comprehensive dataset encompassing clinical and imaging parameters. By identifying critical predictors of early safety outcomes, it seeks to refine patient selection, optimize procedural strategies, and elevate the overall standard of TAVI care [29,30].

## 2. Materials and Methods

### 2.1. Patient Selection

This retrospective, single-centre study was conducted in the Cardiology Department of the Lithuanian University of Health Sciences, Kauno Clinics, from 1 September 2021 to 1 April 2023. The study aimed to evaluate the influence of procedural factors on the outcomes of TAVI in 230 patients diagnosed with severe aortic stenosis. A total of six patients were excluded from the initial pool: four patients declined to participate, one had severe calcification of the aorta, abdominal, and femoral arteries, and one patient was excluded because the TAVI procedure was performed via the subclavian artery.

In the study, 224 patients with severe aortic stenosis were included according to the latest guidelines of the European Society of Cardiology [2]. Inclusion criteria were an older age (≥75 years), previous cardiac surgery, severe frailty, TAVI that is feasible via a transfemoral approach, a porcelain aorta, a high likelihood of severe patient–prosthesis mismatch (AVA < 0.65 cm^2^ m^2^ BSA), severe chest deformation or scoliosis and Heart Team decision for TAVI. Exclusion criteria included elevated myocardial injury markers, other significant heart valvular diseases and any type of aortic aneurysm. The TAVI procedures were performed based on the decisions of the “Heart Team”, which included specialists such as cardiac surgeons, interventional cardiologists, imaging specialists, cardiologists and sometimes other specialists.

Follow-Up: A complete follow-up was conducted over a one-year period, collecting detailed demographic information, medical history, and postoperative outcomes (such as survival rates and hospital readmission frequency). Follow-up was performed through outpatient visits and, when necessary, via telephone contact with patients or their physicians. The follow-up concluded in April 2024. The study was approved by the local ethics committee, and all participants provided written informed consent in accordance with established ethical standards (BE-2-101/08.11.2022).

### 2.2. Data Collection

Echocardiography

All patients underwent blood tests (Hemoglobin, White Blood Cell (WBC), Neutrophil, Lymphocyte, Thrombocyte) and transthoracic echocardiography (TTE) or transoesophageal echocardiography (TEE) both before and after the TAVI procedure. These assessments were conducted 24 h prior to the procedure and 12–48 h post-procedure. TTE or TEE were performed using a Philips echocardiograph (Philips North America, Andover, MA, USA).

The following echocardiographic parameters were included: the left ventricular end-diastolic diameter, the left ventricular end-diastolic diameter index, the septal thickness, the posterior wall thickness, the left ventricle mass, the left ventricular ejection fraction, the left ventricle relative wall thickness, the right ventricle diameter, the right ventricular function, the left atrium diameter, the left atrium volume, the left atrium volume index, the right atrium diameter, the aortic valve annulus diameter, the sinuses of Valsalva diameter, the sinuses of Valsalva index, the ascending aorta diameter, the right ventricle outflow tract acceleration time, the aortic valve maximal velocity, the mean aortic valve gradient, the aortic valve area, the aortic valve area index, the aortic valve velocity ratio, aortic regurgitation severity, mitral regurgitation severity, tricuspid regurgitation severity, tricuspid valve maximal velocity, the tricuspid valve maximal gradient, pulmonary artery systolic pressure, pulmonary artery mean pressure, and the presence of the bicuspid aortic valve.

This detailed echocardiographic assessment allowed for thorough pre- and post-procedure evaluation of cardiac function and valve dynamics in patients undergoing TAVI.

Computed Tomography

All patients underwent contrast-enhanced computed tomography (CT) scans of the heart, aorta, and femoral arteries. The CT scans were performed at Kaunas Clinics using a 640-slice CT scanner with a 0.5 mm slice thickness in 0.275 s per full rotation (Aquilion GENESIS; Canon Medical Systems USA, Inc., Tustin, CA, USA). Each patient received 70–90 mL of Omnipaque Iomeron 400 (Patheon Italia, Ferentino, Italy) contrast agent. After the CT scans, the data were processed using 3Mensio Structural Heart and Vascular software (version 5.1; Pie Medical Imaging, Maastricht, The Netherlands).

The following parameters were evaluated: aortic annulus dimensions (systolic annular aortic perimeter, perimeter-derived diameter, systolic annular aortic area, area-derived diameter, aortic annulus angle), left ventricular outflow tract (LVOT) dimensions (perimeter, area, maximum diameter, minimum diameter, perimeter-derived diameter), sinus of Valsalva (SoV) dimensions (SoV diameters: right coronary sinus, left coronary sinus, non-coronary sinus; right coronary leaflet length; left coronary leaflet length; non-coronary leaflet length), aortic root dimensions (coronary height of the right coronary artery, coronary height of the left coronary artery, Sinotubular junction diameter, Sinotubular junction height, average ascending aorta diameter), calcium quantification (calcium volume), the peripheral arteries’ dimensions (left and right sides: common iliac artery minimum, maximum and average diameters; external iliac artery minimum; maximum and average diameter; femoral artery minimum; maximum and average diameter). These detailed CT measurements allowed for a comprehensive preoperative evaluation of both central and peripheral vascular anatomy to support safe and effective TAVI procedures.

All TAVI procedures were performed under local anesthesia with sedation. Access was obtained through the right or left femoral artery in all patients. The puncture was conducted using the Seldinger technique under ultrasound guidance (Philips North America, Andover, MA, USA) in Doppler mode, targeting either the right or left femoral artery depending on vessel integrity, the absence of atherosclerosis, and significant calcification. Based on CT data, parameters such as the diameter, the bifurcation height of the femoral artery, iliac artery tortuosity, and calcification severity were considered. After puncturing both femoral arteries and placing a 6 French introducer (SuperSheath, Medikit, Tokyo, Japan; Radifocus Introducer IIH, Terumo, Tokyo, Japan), the valve introducer was inserted. In the contralateral femoral artery, a 7 French introducer was placed, and an 8 French introducer was also placed in the femoral vein in the same area. The 6 French introducer was then removed, leaving a soft guidewire in the artery. Following this, a Perclose ProGlide vascular closure device (Abbott Vascular, Santa Clara, CA, USA) was used for vessel closure and securing, after which the Perclose ProGlide was removed, leaving fixed sutures. A soft guidewire was reintroduced to guide the insertion of a 10 French introducer. A 6 French pigtail catheter with a soft guidewire was then advanced through the 10 French introducer to the level of the descending aorta under fluoroscopic guidance, and the guidewire was subsequently removed. A stiff Safari guidewire was then introduced through the pigtail catheter. Once the introducer for the valve was prepared, the pigtail and the 10 French introducer were removed, leaving the Safari guidewire in place. The valve introducer was inserted under fluoroscopic control, and unfractionated heparin (10,000 units) was administered. Invasive arterial pressure monitoring was connected. A bipolar endocardial electrode was inserted into the right ventricle for temporary cardiac pacing through the 8 French introducer in the femoral vein under fluoroscopy. The puncture site was closed using the Perclose ProGlide system (Abbott Vascular, Santa Clara, CA, USA) and an 8F collagen-based Angio-Seal device (Terumo Interventional Systems, Somerset, NJ, USA). The aortic prosthesis was deployed under angiographic and fluoroscopic control. Following valve implantation, aortography was performed to quantify the degree of aortic regurgitation using the Sellers classification, and a follow-up transthoracic echocardiogram was conducted 48 h later.

Two types of valves were implanted in patients: balloon-expandable valves (BEVs) and self-expanding valves (SEVs). The BEVs implanted were Myval (Meril Life Sciences Pvt. Ltd., Mumbai, India) and the SEVs implanted were CoreValve/Evolut R/Evolut Pro (Medtronic, Minneapolis, MN, USA) and Acurate Neo 2 (Boston Scientific Corp., Watertown, MA, USA).

Clinical outcomes and adverse events were documented at 30 days post-procedure. Follow-up was conducted through phone calls with all patients, as well as regular monitoring via the electronic medical portal eSveikata.lt to ensure the thorough tracking of patient progress and potential complications over time.

Early safety outcomes for 30 days were defined according to the Valve Academic Research Consortium II (VARC-2) criteria [16]. The following early safety outcomes were included: all course mortality, stroke, life-threatening bleeding, valve-related dysfunction requiring a repeat procedure, and acute kidney injury—stage 2 or 3. A permanent pacemaker was implanted if an advanced atrioventricular block developed, following the European Society of Cardiology (ESC) guidelines for patients with an acquired AV block in specific clinical situations.

### 2.3. Model Selection

To solve the issue of an imbalanced dataset, adaptive synthetic (ADASYN) sampling was used, which generates synthetic instances, particularly focusing on those that are difficult to learn [17]. This approach could be considered as a generalization of the Synthetic Minority Over-sampling Technique (SMOTE) by better aligning the generated samples with the underlying distribution of the minority class.

A random forest classifier (RF) was used to build a classification model to predict early safety outcomes after TAVI. The selection of RF over other machine learning approaches is attributed to its several advantages such as its robustness to overfitting, resilience to noisy data, ability to handle non-linear relationships, demonstrating a good performance with a large number of features, and its well-handling of imbalance data when combined with techniques like SMOTE and ADASYN [18,19,20]. Furthermore, the RF model was tuned via a grid-search algorithm for optimal hyperparameters and validated using a 10-fold stratified cross-validation. The discriminatory power of built machine learning models was determined using confusion matrix and performance measures such as accuracy, precision, recall, and F1-score [21].

Finally, to understand how an individual feature contributes to the prediction of early safety outcomes, SHapley Additive exPlanations (SHAPs) were calculated. More specifically, the SHAP value is determined via measuring the average marginal contribution of a feature value across all potential feature combinations [22,23].

Model performance.

The original data sample was split into training and testing with a ratio 75:25. As a result, 56 unseen observations were reserved to test the predictions for the imbalanced case.

ADASYN sampling with a sampling strategy of α=0.9 and k=5 nearest neighbours was used to balance the training data sample, consisting of 245 observations.

A grid-search was performed to fine-tune the hyperparameters of random forest using 10-fold cross validation. As a result, Table 1 and Table 2 present the discriminatory power of fitted random forest with fine-tuned parameters: criterion = “Gini” to measure the quality of split, the maximum depth of the tree is 8, and the maximum number of features to consider when looking for the best split is $\sqrt{number\;of\;features}$ , number of trees in the forest = 300.

## 3. Results

Between 2021 and 2023, a total of 224 patients with severe aortic stenosis were included in the study following the Heart Team’s decision to undergo TAVI. The collected patients were divided into two groups: those with early clinical outcomes and those without. The mean age of patients without early clinical outcomes was 79.96 ± 6.97 years, while for patients with outcomes, it was slightly higher at 81.94 ± 3.38 years. However, this difference was not statistically significant (*p* = 0.251). In the group without early clinical outcomes, 39.4% were men and 60.6% were women. In the group with early clinical outcomes, the distribution was similar: 38.8% men and 61.2% women.

Out of 224 patients, 23 (10.3%) had previously undergone cardiac surgery. Coronary artery disease (CAD) and prior percutaneous coronary intervention (PCI) also did not demonstrate significant differences between the groups (*p* = 0.432 and *p* = 0.452, respectively). However, a history of myocardial infarction (MI) was significantly associated with early clinical outcomes: 32.0% of patients in the outcomes group compared to 68.0% in the group without outcomes (*p* = 0.049). The EuroScore II was higher in the group with early clinical outcomes (7.3 ± 6.61%) compared to the group without outcomes (4.9 ± 3.55%), with borderline significance (*p* = 0.059). Patients with NYHA class III-IV were more likely to experience early clinical outcomes compared to those with NYHA class I-II, but the differences did not reach statistical significance (*p* = 0.355). Baseline echocardiographic parameters, such as the left ventricular end-diastolic dimension (LVEDd) and left ventricular ejection fraction (LVEF), were similar between the groups. Other parameters, such as the mean gradient across the aortic valve (AV Gmean) and pulmonary artery systolic pressure (PASP), also did not show significant differences between the groups.

CT scan data revealed that the aortic valve calcification volume (AVCV) was significantly higher in patients with early clinical outcomes (*p* = 0.025). Other CT parameters, such as aortic valve diameter (AVd) and perimeter-derived diameter (AVp.d), were slightly higher in the outcomes group but did not reach statistical significance (*p* = 0.075 and *p* = 0.104, respectively) (Table 3).

**Figure 1:**
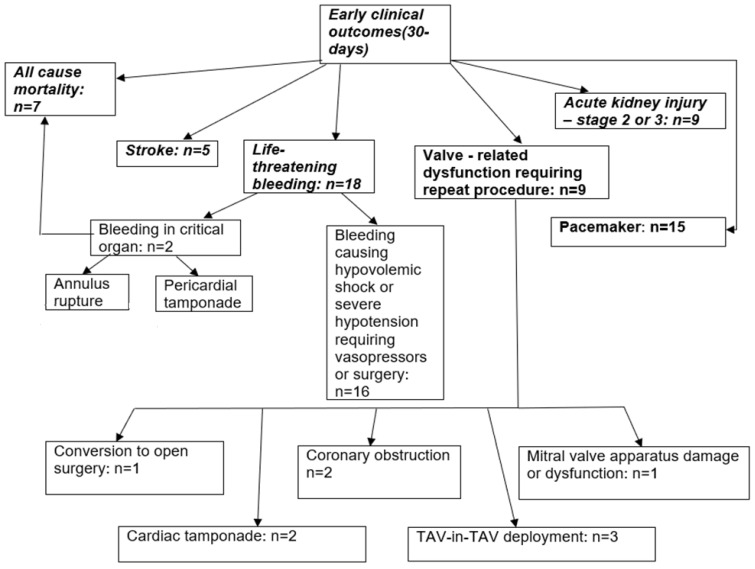
Early safety outcome characteristics.

In the group with early clinical outcomes, definitions were categorized based on recommendations (VARC II), specifying composite endpoints referred to as “early safety outcomes after TAVI within 30 days”. Early safety outcomes after TAVI within 30 days were observed in 49 patients (21.8%). Among them, 25 patients had 1 outcome, and 24 patients had 2 or more outcomes. All-cause mortality was recorded in seven cases (14.3%). Stroke occurred in five patients (10.2%). Life-threatening bleeding was observed in 18 patients (36.7%), including 16 cases requiring vasopressors or surgery. Among these were one case of conversion to open surgery, two cases of coronary obstruction, and two cases of cardiac tamponade. Valve dysfunction was reported in nine patients (18.4%), including three cases requiring TAV-in-TAV implantation. Acute kidney injury (stage 2 or 3) was diagnosed in nine patients (18.4%). Pacemaker implantation was performed in 15 patients (30.6%) (Figure 1).

**Figure 2:**
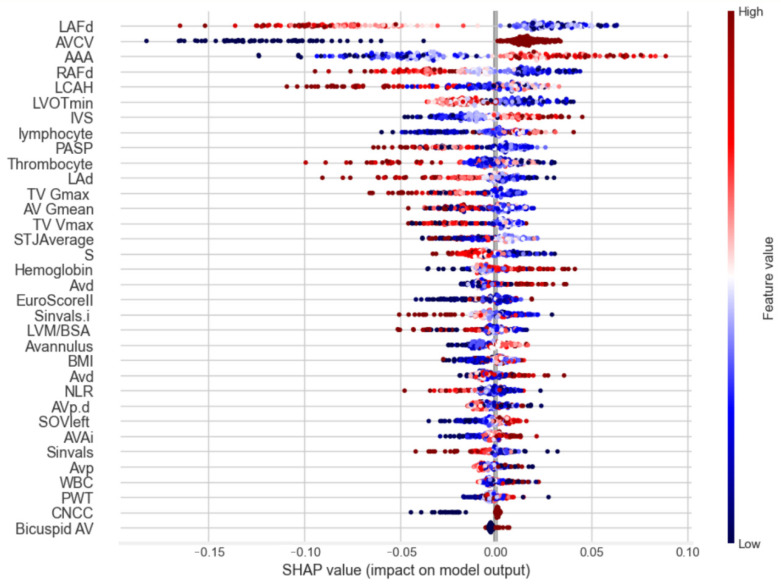
Feature contribution to the prediction of early safety outcomes in patients undergoing TAVI. (AAA—angle of aortic annulus; AV Gmean—mean aortic valve gradient; AVAi—aortic valve area index; AVCV—aortic valve calcified volume; AVd—aortic valve diameter; AVp—aortic valve perimeter; AVp.d—aortic valve perimeter derived; BMI—Body Mass Index; Bicuspid AV—bicuspid aortic valve; CNCC—calcified non-coronary cusp; EuroScoreII—European System for Cardiac Operative Risk Evaluation II; Hemoglobin—Hemoglobin; IVS—Interventricular Septum; LAd—left atrium diameter; LAFd—left femoral artery diameter; LCAH—left coronary artery height; LVM/BSA—Left Ventricular Mass/Body Surface Area; LVOTmin—left ventricular outflow tract minimal size; Lymphocyte—Lymphocyte Count; NLR—Neutrophil-to-Lymphocyte Ratio; PASP—pulmonary artery systolic pressure; PWT—posterior wall thickness; RAFd—right femoral artery diameter; S’—right ventricular function; Sinvals—sinuses of Valsalva; Sinvals.i—sinuses of Valsalva index; SOVleft—Sinus of Valsalva Left Side; STJAverage—sinotubular junction average; Thrombocyte—Thrombocyte Count; TV Gmax—maximal tricuspid valve gradient; TV Vmax—tricuspid valve maximal velocity; WBC—White Blood Cell Count).

To identify key factors influencing early safety outcomes following transcatheter aortic valve implantation (TAVI), the SHapley Additive Explanations (SHAPs) method was applied. This analysis provided an in-depth understanding of the contribution of each feature to the model’s predictions, highlighting several key predictors. SHAP analysis demonstrated that the left femoral artery diameter, a higher aortic valve calcification volume and a larger angle of the aortic annulus were associated with poorer early safety prognoses (Figure 2).

The SHAPs chart (Figure 2) demonstrates the ranked influence of predictors on the model’s outcomes. Features with high SHAP values exerted the most significant impact on predictions. The colour gradients (red for high feature values, blue for low) illustrate the directional effects of each predictor on the model’s output.

## 4. Discussion

The findings from this study emphasize the potential of machine learning (ML) models in predicting early safety outcomes following transcatheter aortic valve implantation (TAVI). Specifically, the random forest model demonstrated its ability to integrate diverse clinical and imaging data, identifying significant predictors of adverse outcomes such as left femoral artery diameter and aortic valve calcification volume.

The association between a smaller left femoral artery diameter and vascular complications aligns with the existing literature, highlighting its role as a critical predictor. Studies have shown that a restricted femoral artery diameter increases procedural difficulty and the risk of vascular injuries. Narrow vascular access is often associated with procedural challenges and can lead to higher rates of bleeding or vascular rupture. Addressing these anatomical variations with precise preprocedural planning can mitigate risks, as noted in studies on pre-TAVI imaging protocols [31,32].

Additionally, a higher aortic valve calcification volume was strongly linked to adverse outcomes such as paravalvular regurgitation and valve dysfunction. This finding supports previous research advocating for precise preoperative imaging to assess calcification and optimize procedural strategies. Extensive calcification has been shown to compromise prosthetic valve deployment and functionality, emphasizing the need for innovative devices that adapt to calcified anatomies [33].

Moreover, patients with elevated pulmonary artery pressures were at increased risk of poor outcomes. This relationship underscores the importance of assessing hemodynamic parameters, as pulmonary hypertension is a known prognostic factor in TAVI. Elevated pressures can indicate pre-existing right heart strain, potentially complicating postoperative recovery [34]. Incorporating pulmonary artery pressure monitoring into patient evaluation workflows has been suggested to enhance risk stratification [35].

The use of adaptive synthetic sampling (ADASYN) sampling in this study to address data imbalance proved effective, improving the model’s predictive accuracy and robustness. Balancing techniques like ADASYN sampling are particularly valuable in medical datasets, where under-represented outcomes often challenge predictive reliability. Other approaches, such as SMOTE and ensemble learning techniques, have similarly demonstrated success in addressing imbalances in TAVI-related data [36,37].

Technological advancements in TAVI devices, including balloon-expandable and self-expanding prostheses, also influenced outcomes. The selection of the appropriate prosthesis type, tailored to individual patient anatomy, has been shown to minimize complications [38]. Comparative studies have indicated varying rates of paravalvular leakage and durability between device types, underscoring the importance of individualized device selection [17,39].

Shapley Additive Explanations (SHAPs) analysis, employed in this study, provided transparency in understanding the model’s predictions. SHAPs effectively identified critical features such as left femoral artery diameter and aortic valve calcification, enhancing interpretability and clinical applicability. Similar explainable AI techniques have been validated in other cardiac intervention studies, highlighting their value in clinical decision-making [20].

Another important factor is the impact of comorbidities such as chronic kidney disease and coronary artery disease on early outcomes. Chronic kidney disease has been associated with increased risks of contrast-induced nephropathy during TAVI, necessitating careful patient selection. Similarly, coronary artery disease often requires concomitant interventions, which can complicate procedural outcomes.

Finally, the integration of multimodal imaging, including echocardiography and CT, with ML algorithms offers promising avenues for improving TAVI outcomes. Combining preoperative imaging with ML can refine risk models and enhance patient stratification. Recent advancements in fusion imaging, integrating CT and 3D echocardiography, have shown promise in reducing procedural errors [35].

## 5. Conclusions

This study highlights the effectiveness of machine learning (ML) models in predicting early safety outcomes in TAVI procedures. Using a random forest model with ADASYN sampling, the research identified key predictors such as a reduced femoral artery diameter and increased aortic valve calcification volume, both associated with a higher risk of complications. Future studies should expand on these findings by incorporating larger datasets, evaluating novel device designs, and exploring advanced ML techniques to further refine predictive accuracy and clinical utility.

## Figures and Tables

**Table 1:** Confusion matrix: cross-validation testing for balanced sample.

		Predicted outcome		
		0	1	Accuracy = 0.8571 Precision = 0.9 Recall = 0.7826 F1-score = 0.8372
Known outcome	0	24	2	
	1	5	18	

**Table 2:** Confusion matrix: cross-validation testing for imbalanced sample with threshold = 0.4.

		Predicted outcome		
		0	1	Accuracy = 0.8571 Precision = 0.6429 Recall = 0.75 F1-score = 0.6923
Known outcome	0	39	5	
	1	3	9	

**Table 3:** Preprocedural baseline characteristics before TAVI.

Variables	Early Clinical Outcomes (No)	Early Clinical Outcomes (Yes)	*p*-Value
Gender:			0.934
Male	69 (39.4%)	19 (38.8%)	
Female	106 (60.6%)	30 (61.2%)	
Age (years), mean ± SD	79.96 ± 6.97	81.94 ± 3.38	0.251
BMI (kg/m^2^), mean ± SD	28.94 ± 6.22	28.88 ±6.48	0.974
AH	165 (78.2%)	46 (21.8%)	0.914
DM	46 (79.3%)	12 (20.7%)	0.800
CAD	157 (78.9%)	42 (21.1%)	0.432
Previous MI	34 (68.0%)	16 (32.0%)	0.049
CABG	16 (69.6%)	7 (30.4%)	0.295
PCI	173 (77.9%)	49 (22.1%)	0.452
EuroScore II (%), mean ± SD	4.9 ± 3.55	7.3 ± 6.61	0.059
NYHA class:			0.355
1–2 class	55 (82.3%)	11 (17.7%)	
3–4 class	124 (76.5%)	38 (23.5%)	
Echocardiographic findings before TAVI			
LVEDd (mm), mean ± SD	48.2 ± 5.6	46.94 ± 7.66	0.452
LV EF (%), mean ± SD	46.9 ± 11.98	44.94 ± 13.87	0.565
S’, mean ± SD	11.37 ± 2.89	10.9 ± 3.2	0.561
PASP, mean ± SD	46.53 ± 15.52	41.49 ± 10.52	0.204
Bicuspid AV	12 (80.0%)	3 (20.0%)	0.856
AVA (mm^2^), mean ± SD	0.76 ± 0.20	0.81 ± 0.22	0.369
AVAi, mean ± SD	0.41 ± 0.11	0.44 ± 0.12	0.423
AV Gmean, mmHg, mean ± SD	48.38 ± 18.6	42.1 ± 11.48	0.176
AR	76 (82.6%)	16 (17.4%)	0.175
Sinvals.i, mean ± SD	18.75 ± 2.99	18.72 ± 3.55	0.969
TV Vmax, mean ± SD	3.08 ± 0.6	0.92 ± 0.43	0.284
TV Gmax, mean ± SD	39.63 ± 15.42	34.88 ± 10.52	0.229
TR	107 (78.7%)	29 (21.3)	0.804
LA diameter, mean ± SD	45.41 ± 5.1	44.27 ± 5.44	0.421
MSCT findings			
AVd, mean ± SD	24.68 ± 2.23	25.86 ± 2.91	0.075
AVp.d, mean ± SD	24.87 ± 2.23	25.95 ± 2.87	0.104
AVCV:			0.025
1.	57 (87.7%)	8 (12.3%)	
2.	117 (74.1%)	41 (25.9%)	
AVp, mean ± SD	78.2 ± 7.01	81.55 ± 8.96	0.105
AAA, mean ± SD	49.82 ± 7.96	54.0 ± 11.48	0.089
LCAH, mean ± SD	13.96 ± 3.40	13.93 ± 2.47	0.976
CNCC	154 (77.8%)	44 (22.2%)	0.800
RCAH, mean ± SD	16.17 ± 3.49	17.32 ± 3.21	0.222
LVOT min, mean ± SD	21.3 ± 2.88	21.82 ± 2.92	0.509
STJ Average, mean ± SD	31.82 ± 25.61	30.56 ± 3.39	0.836
RAFd, mean ± SD	8.14 ± 1.21	8.01 ± 1.63	0.714
LAFd, mean ± SD	7.76 ± 1.05	7.96 ± 1.38	0.508
Blood test			
Hemoglobin, mean ± SD	120.8 ± 13.91	118.66 ± 13.10	0.569
WBC, mean ± SD	6.25 ± 1.57	7.12 ± 2.41	0.068
Thrombocyte, mean ± SD	205.65 ± 65.97	209.50 ± 58.70	0.826

(AH—arterial hypertension; DM—diabetes mellitus; CAD—coronary artery disease; Previous MI—previous myocardial infarction; CABG—coronary artery bypass grafting; Previous PCI—percutaneous coronary intervention; EuroScore II—European System for Cardiac Operative Risk Evaluation II; NYHA—New York Heart Association; BMI—Body Mass Index; LVEDd—left ventricular end-diastolic diameter; LVEF—left ventricle ejection fraction; S’—right ventricular function; PASP—pulmonary artery systolic pressure; Bicuspid AV—bicuspid aortic valve; AVA—aortic valve area; AVAi—aortic valve area index; AV Gmean—mean aortic valve gradient; AR—aortic regurgitation; Sinvals.i—sinuses valsalva index; TV Vmax—tricuspid valve maximal velocity; TV Gmax—maximal tricuspid valve gradient; TR—tricuspid regurgitation; LAd—left atrium diameter; AVd—aortic valve diameter; AVp.d—aortic valve perimeter derived; AVCV—aortic valve calcified volume; AVp—aortic valve perimeter; AAA—angle of aortic annulus; LCAH—left coronary artery height; RCAH—right coronary artery height; CNCC—calcified non-coronary cusp; LVOTmin—left ventricular outflow tract minimal size; STJAverage—sinotubular junction average; RAFd—right femoral artery diameter; LAFd—left femoral artery diameter).

## Data Availability

The data supporting this study are not publicly available due to ethical restrictions. Access to the data may be granted upon reasonable request and is subject to approval by the local ethics committee.

## Abbreviations

The following abbreviations are used in this manuscript:

AS	Aortic stenosis
AV	Aortic valve
BSA	Body surface area
BEVs	Balloon-expandable valves
CABG	Coronary artery bypass graft
CT	Computed tomography
CAD	Coronary artery disease
EuroSCORE II	European System for Cardiac Operative Risk Evaluation
LVEF	Left ventricular ejection fraction
MI	Myocardial infarction
MR	Mitral valve regurgitation
NYHA	New York Heart Association
PCI	Percutaneous coronary intervention
SAVR	Surgical aortic valve replacement
SEV	Self-expandable valve
SPSS	Statistical analysis was performed using SPSS
TAVI	Transcatheter aortic valve implantation
VARC-2	Valve Academic Research Consortium II
